# Novel myeloma patient-derived xenograft models unveil the potency of anlotinib to overcome bortezomib resistance

**DOI:** 10.3389/fonc.2022.894279

**Published:** 2022-08-05

**Authors:** Yanhua Yue, Yang Cao, Xunyuan Mao, Fei Wang, Peng Fan, Long Qian, Shuxin Guo, Feng Li, Yanting Guo, Tongbing Chen, Yan Lin, Weimin Dong, Yue Liu, Yuhui Huang, Weiying Gu

**Affiliations:** ^1^ Department of Hematology, The First People’s Hospital of Changzhou, Third Affiliated Hospital of Soochow University, Changzhou, China; ^2^ Cyrus Tang Hematology Center, Collaborative Innovation Center of Hematology, State Key Laboratory of Radiation Medicine and Prevention, Soochow University, Suzhou, China; ^3^ Department of Pathology, The First People’s Hospital of Changzhou, Third Affiliated Hospital of Soochow University, Changzhou, China

**Keywords:** multiple myeloma, patient-derived xenograft, anlotinib therapy, tumor angiogenesis, tumor-associated macrophages

## Abstract

Multiple myeloma (MM) remains a common hematologic malignancy with a 10-year survival rate below 50%, which is largely due to disease relapse and resistance. The lack of a simple and practical approach to establish myeloma patient-derived xenograft (PDX) hampers translational myeloma research. Here, we successfully developed myeloma PDXs by subcutaneous inoculation of primary mononuclear cells from MM patients following series tumor tissue transplantations. Newly established myeloma PDXs retained essential cellular features of MM and recapitulated their original drug sensitivities as seen in the clinic. Notably, anlotinib therapy significantly suppressed the growth of myeloma PDXs even in bortezomib-resistant model. Anlotinib treatments polarized tumor-associated macrophages from an M2- to an M1-like phenotype, decreased tumor vascular function, and accelerated cell apoptosis in myeloma PDXs. Our preclinical work not only unveiled the potency of anlotinib to overcome bortezomib resistance, but also provided a more practical way to establish MM PDX to facilitate myeloma research.

## Introduction

Multiple myeloma (MM) is a plasma cell malignancy of the hematopoietic system (1). The advents of novel treatments have changed the management of myeloma and extended overall survival (2–7). Again, chimeric antigen receptor T-cell (CAR-T) therapy is recently emerging as a promising treatment option for relapsed and resistant MM (7). However, MM is still accompanied by repeated relapse and resistance with a 10-year survival rate below 50% (8, 9). Therefore, new strategies to overcome myeloma recurrence and resistance remain much anticipated.

Patient-derived xenografts (PDXs) have appeared as an important platform to develop new treatment strategies and to identify new biomarkers in oncology. Primary myeloma cells from the majority of patients do not propagate in severe combined immunodeficiency (*SCID*) mice. Previous studies revealed that mobilized blood mononuclear cells or CD34-enriched cells from patients with advanced disease could develop myeloma in irradiated nonobese diabetic/SCID (*NOD/SCID*) mice by intracardiac injection (10–12). The intracardiac injection in mice is technically difficult, which limits its wide applications. Subsequently, another study developed an *in vivo* system for primary human myeloma by rabbit bone implantation (13). Although this model can mimic typical manifestations of clinical myeloma, this modeling process is very complicated and expensive. Nowadays, PDXs of MM are mainly established by intravenous or intra-osseous injection with primary myeloma cells (10, 13–15), but the success rates are very low. Thus, simple and practical approaches to develop myeloma PDX are urgently needed to conduct translational and mechanistic studies of MM.

MM is initiated in the bone marrow (BM) microenvironment and increased angiogenesis facilitates MM progression. Important proangiogenic factors, produced by BM stromal cells and plasma cells, stimulate angiogenesis (16, 17). An enhanced vascular formation has been considered as an essential feature of MM (18). Therefore, antiangiogenic therapy targeting these proangiogenic signaling pathways could be used to control MM. Anlotinib is a novel multi-targeted receptor tyrosine kinase inhibitor that targets vascular endothelial growth factor receptor (VEGFR) 1-3, c-Kit, platelet-derived growth factor receptor (PDGFR)-α/β, and fibroblast growth factor receptor (FGFR) 1-4. Moreover, anlotinib exhibits anti-MM activity in NCI-H929 myeloma cell line-derived xenografts (19). Hence, we aimed to test the efficacy of anlotinib in myeloma PDXs.

In this study, we developed a simple and feasible way to establish myeloma PDX and successfully set up four myeloma PDXs, one of which was bortezomib-resistant. The morphological, phenotypic and drug-sensitive characteristics of bortezomib-resistant myeloma PDX recapitulated the properties of the MM patient. Furthermore, our data showed that anlotinib treatments significantly inhibited tumor growth of bortezomib-resistant myeloma and induced cell apoptosis. Anlotinib treatments reduced tumor vascular function, increased tumor-associated macrophages (TAMs), and polarized M2- to M1-like phenotype in MM PDXs. These results suggest that subcutaneous myeloma PDX could be a feasible and practical strategy to explore novel therapies for MM, and anlotinib is a promising therapy for relapsed and resistant MM.

## Methods

### Cell line and patient samples

Human MM cell line MM.1S was obtained from the American Type Culture Collection (Manassas, VA, USA). Cells were cultured in RPMI 1640 medium supplemented with 100 IU ml^-1^ penicillin, 100 μg ml^-1^ streptomycin, and 10% fetal bovine serum (Hyclone) at 37°C in 5% CO_2_ atmosphere in a humidified chamber. BM cells and pleural effusion cells from MM patients were collected after written informed consent and the institutional ethics committee approval by The Third Affiliated Hospital of Soochow University. According to the International Myeloma Working Group, diagnosis and relapse of MM were defined (20, 21). A total of fifteen patient BM samples were used in this study. Nine of them were newly diagnosed with MM, while six patients were relapsed. Another one extramedullary myeloma pleural effusion sample came from one patient with relapsed and resistant MM. Mononuclear cells (MNCs) were isolated from BM and pleural effusion samples using Ficoll-Hypaque (Sigma-Aldrich).

### Patient-derived xenograft models

*NOD.CB17-Prkdc^scid^ Il2rg^tm1^/Bcgen (B-NDG)* female mice (6-weeks old) were purchased from BIOCYTOGEN (Beijing, China). All animal experiments were approved by the Animal Research Committee of The Third Affiliated Hospital of Soochow University. Mice were kept in the specific pathogen-free gnotobiotic animal facility at Soochow University. MM.1S cells (2 × 10^6^ cells) or MNCs (≥2 × 10^6^ cells, according to sample size) from MM patients were mixed with 50% Matrigel (BD Biosciences) and then inoculated subcutaneously into the flanks of *NDG* mice. Four PDXs were established, one of them is bortezomib-resistant. **Table S1** showed the characteristics of the four patients. Tumor fragments from PDXs were transplanted into other *NDG* mice in less than six generations. When tumors reached 6-8 mm in diameter, tumor tissues were isolated and prepared for single-cell suspension, hematoxylin-eosin (HE) and immunohistochemical (IHC) analysis. In treatment experiments, when tumor diameter reached 4-6 mm, mice were randomly assigned to treatment or control groups. In the treatment groups, mice were administered with different doses of anlotinib orally daily, 0.5 mg/kg bortezomib intraperitoneally twice a week, 25 mg/kg lenalidomide orally five times a week or 1 mg/kg dexamethasone intraperitoneally daily for about two weeks. The sizes of tumors were measured every 3 days, and the tumor volume was estimated by the formula [(long axis) × (short axis)^2^/2]. When mice were euthanized, tumor tissues were isolated, weighed and prepared for flow cytometric analysis and tissue vessel analysis. Meanwhile, the colon tissues were also isolated and prepared for tissue vessel analysis.

Anlotinib, bortezomib, lenalidomide, and dexamethasone were gifts from Jiangsu Chia-Tai Tianqing Pharmaceutical Co, Ltd (Nanjing, China). Anlotinib, lenalidomide, and dexamethasone were dissolved with double distilled water (ddH_2_O), 0.5% methyl cellulose, and phosphate-buffered saline (PBS), respectively. Bortezomib was firstly dissolved in Dimethyl Sulfoxide (Sigma-Aldrich), then the stock solution of bortezomib was diluted by ddH_2_O.

### HE and IHC staining

The isolated tumors were fixed and prepared to perform HE and IHC analysis. After antigen retrieval, IHC staining was conducted using primary antibodies **[**mouse anti-human CD138 (MAB-0200, MI15), mouse anti-human CD38 (MAB-0755, MX044), mouse anti-human Ki-67 (MAB-0672, MX006)] from MXB Biotechnologies (Fuzhou, China), and the secondary antibody goat anti-mouse IgG with an alkaline phosphatase-linker antibody conjugate system (SAP-9100) from Beijing Zhong Shan Golden Bridge Biological Technology (Beijing, China). Two investigators independently evaluated HE and IHC staining.

### Single-cell suspension preparation

The isolated tumor tissues were minced and digested at 37°C for 45 min with cell detach solution. The digested mixtures were ground and filtered with 70-μm cell strainers. The whole process of single-cell preparation was carried out under sterile conditions. The culture conditions of single-cell were the same as that of MM.1S. CD138^+^ myeloma cells were enriched from the single-cell suspension of PDXs by Human CD138 MicroBeads (Miltenyi Biotech).

### Flow cytometric analysis

Single-cell suspension was prepared in cold flow buffer (1% bovine serum albumin, 0.1% NaN3 in PBS). Samples were examined by a Gallios flow cytometer (Beckman) and data were analyzed with Kaluza software (version1.3). Anti-mouse monoclonal antibodies [CD45-BV421 (103134, 30-F11), CD45-PE (12-0451-83, 30-F11), CD11b-BV510 (101263, M1/70), Ly-6G-FITC (551460, 1A8), Gr1-APC-Cy7(47-5931-82, RB6-8C5), F4/80-PE (12-4801-82, BM8), CD11c-PE-Cy7 (25-0114-82, N418), CD206-APC (141708, C068C2), CD86-FITC (11-0862-81, GL-1)] and anti-human monoclonal antibodies [CD138- BV421 (562935, MI15), CD38-PE-Cy7 (25-0389-41, HIT2), kappa-FITC (643773, TB28-2), and lambda-PE (642919, 1-155-2)] were used.

### Cell viability assay

Myeloma cells were seeded in 96-well plates and treated with anlotinib at the indicated concentrations. According to the manufacturer’s instructions, cell viability was assessed by a Cell Counting Kit-8 (CCK-8, Beyotime Biotechnology). Cell viability was calculated by [cell viability rate (%) = (administration group value - negative control group value)/(non-administration group value – Negative control group value) × 100%].

### Western blotting

Cells were harvested, washed, and lysed for total protein extraction. Equal quantities of protein extract were injected into sodium dodecyl sulfate–polyacrylamide gel and transferred to polyvinylidene difluoride (PVDF) membranes. After blocking with 5% non-fat milk, the membranes were incubated with primary antibodies overnight at 4°C, followed by incubation with alkaline phosphatase-conjugated secondary IgG antibody. The bands were incubated with a DAB kit and analyzed with an imaging system. Antibodies against β-actin (#AC026) and c-Myc (#A19032) were purchased from ABclonal (Wuhan, China).

### Tumor vessel perfusion analysis

Tumor blood vessel analysis was performed as previously described (22–24). Briefly, 5 minutes after intravenous injection of Hoechst 33342 (Ho33342, 10 mg/kg in 200 μl PBS, Sigma-Aldrich), tumor tissues were isolated and prepared to incubate with the primary antibody (anti-CD31, 550274, MEC13.3, BD Biosciences) at 4°C and the secondary antibody (Alexa Fluor 647 goat anti-Armenian Hamster, 127–605-160, Jackson ImmunoResearch) at room temperature for 2 h. Cell nuclei staining (Sytox Green, S7020, Molecular Probes) was applied to counterstain the slides. Fluorescent images of tumors were collected using an Olympus FV3000 confocal laser-scanning microscope. A 20× objective collected 640 × 640 μm tiles, and an automated stage scanned through the entire cross section of tumor tissue. The imaged tiles were stitched into a final mosaic image by an Olympus FV3000 software. Five photographic areas, excluding the tumor periphery, were randomly taken from every tumor tissue (640 × 640 μm^2^). Mean fluorescence intensity (MFI) of CD31 positivity and Ho33342 stained areas were quantified by Image-Pro Plus software (version 6.0). The vascular function of colon tissues was analyzed as the above.

### Cell cycle by flow cytometry and TUNEL staining

For cell cycle distribution, single-cell suspension was incubated with DNA Staining solution and permeabilization solution (MultiSciences) for 30 min at room temperature, and then examined through flow cytometry. Tumor tissue slices were permeabilized with 0.5% Triton X-100 and incubated with TUNEL detection solution (Beyotime Biotechnology) for 60 min and then DAPI for 5 min at 37°C. Five photographic areas of every slice were randomly taken by an Olympus FV3000 confocal laser-scanning microscope. The MFI of TUNEL positivity was calculated by Image-Pro Plus software (version 6.0).

### Statistics

Statistical analysis was conducted using Prism software (version 7, GraphPad). The quantitative data are presented as the mean ± standard deviation (SD). The differences between two groups were analyzed using two-tailed Student’s *t*-test. The half-maximal inhibitory concentration (IC50) was calculated using the dose-response curve using SPSS version 23 software (IBM Corporation, Chicago). *P*<0.05 was considered as statistically significant.

## Results

### The morphological and phenotypic features of tumor cells from myeloma PDX

**Figure 1:**
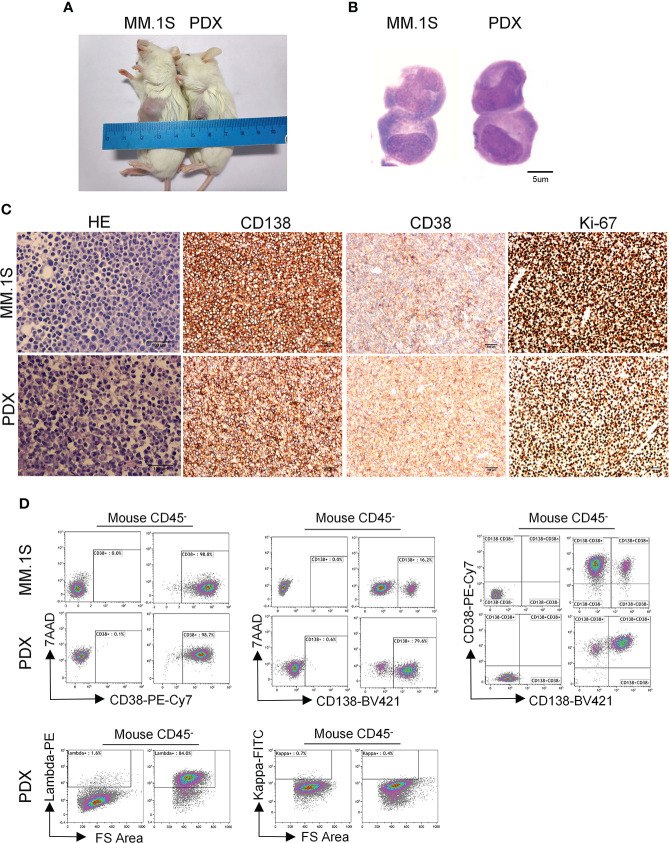
Morphological and phenotypic features of tumor cells from newly established myeloma PDX. MM.1S cells and mononuclear cells isolated from BM and pleural effusion samples of MM patient were inoculated subcutaneously in *NDG* mice. When tumors reached 6-8 mm in diameter, tumors were removed and prepared for HE staining, IHC staining, and single-cell suspensions. **(A)** Representative image of myeloma MM.1S and PDX models. **(B)**The morphology of tumor cells from myeloma MM.1S and PDX models by Giemsa staining. Scale bar: 5 μm. **(C)** Representative IHC stainings of tumor tissues. Scale bar: 100 μm. **(D)** The single-cell suspensions were analyzed by flow cytometry. The doublet or aggregated events were gated out according to side scatter area (SSC-A) and side scatter width (SSC-W). 7-AAD staining was used to gate out dead cells. The expression levels of CD138, CD38, kappa, and lambda were analyzed in mouse CD45^-^ cells. Myeloma cell line MM.1S was used as a positive myeloma cell control. The left panels of flow charts were isotype controls. PDX, patient-derived xenograft; HE, hematoxylin-eosin; IHC, immunohistochemical.

MM is a hematologic malignancy with a high risk of resistance and relapse. Currently, myeloma PDX is generally developed in immunodeficient mice using rabbit bone graft, and then primary myeloma cells were injected into rabbit bone cavity (13). The procedure is complicated and difficult with a low success rate (25). To explore a more practical approach to develop myeloma PDX, we subcutaneously inoculated MNCs from BM or pleural effusion of MM patients into *NDG* mice. When primarily inoculated tumors grew to 6-8 mm in diameter, tumor tissues were removed and cut into small pieces. The small tumor tissue pieces were subcutaneously transplanted into new *NDG* mice. After 3-4 times of transplantations, MM PDX tumors grew at a steady rate and can be used for the following experiments (**Figure 1A**).

To characterize the features of newly established myeloma PDX, we collected tumor tissues from one PDX and obtained single-cell suspension to perform Giemsa, HE, and IHC staining. The Giemsa and HE staining showed that most tumor cells from this PDX had a malformed plasma cell morphology with eccentric nuclei, amphophilic cytoplasm and a perinuclear halo of clearer cytoplasm, which were comparable with those of MM.1S, a commonly used MM cell line (**Figures 1B, C**). MM cells from patients commonly express CD138, CD38, and restrictively express light chains of kappa or lambda (26). Thus, we carried out IHC staining to analyze the expression of CD138, CD38, and Ki-67 in the PDX and MM.1S tumors. MM.1S cells positively expressed CD138 and CD38 and about 90% of them were Ki-67^+^ (**Figure 1C**). Similarly, tumor cells from the myeloma PDX expressed both CD138 and CD38 and 70% of them were Ki-67^+^ (**Figure 1C**). Therefore, the PDX exhibited the phenotype of CD138^+^ malignant plasma cells. To confirm the phenotypic characteristics, we performed flow cytometric analysis of MM.1S cells and tumor cells from the PDX. Most of MM.1S cells expressed CD138 and CD38, and the majority of tumor cells from the PDX were also CD138^+^ CD38^+^ (**Figure 1D**). As to the light chains, tumor cells from the myeloma PDX restrictively expressed lambda without the expression of kappa (**Figure 1D**). Collectively, the phenotypic features of tumor cells from the PDX were CD138^+^CD38^+^lambda^+^kappa^-^. Therefore, tumor cells from our established PDX possess the essential morphological and phenotypic features of MM cells.

### The proliferation characteristics of myeloma PDX

**Figure 2:**
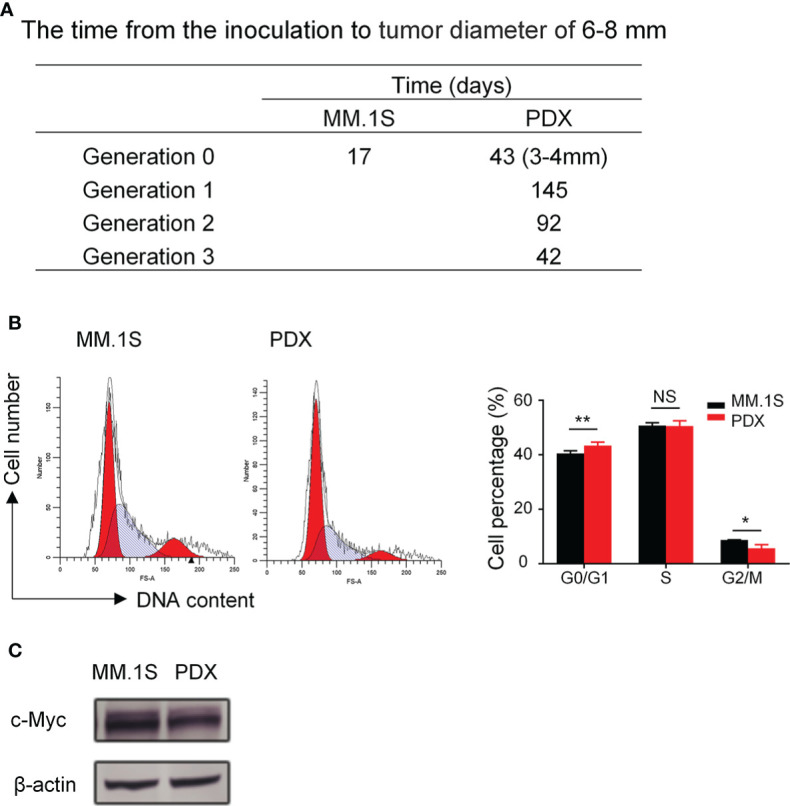
Myeloma PDX tumors grew slower than MM.1S-derived tumors. The first generation of myeloma PDX was established in *NDG* mice as described in **Figure 1**. When the PDX tumor reached 6-8 mm in diameter, tumor tissues were removed and tumor fragments were transplanted to new *NDG* mice. The single-cell suspension for cell cycle analysis was acquired from removed tumors. MM.1S cells (2×10^6^ cells) were inoculated subcutaneously into the flank of *NDG* mice. **(A)** The time (days) from the inoculation to tumor diameter reaching 6-8 mm in MM.1S and PDX myeloma mouse models. **(B)** Cell cycle of MM.1S cells and tumor cells from myeloma PDX was analyzed by flow cytometry. **(C)** MM.1S and PDX whole cell lysates were subjected to western blotting using antibodies against c-Myc and β-actin. PDX: patient-derived xenograft. Significance was determined by unpaired two-tailed Student’s *t*-test. Each experiment was performed in triplicate and repeated at least three times. All data were presented as means ± SD. *NS*, no significance, *P < 0.05, **P < 0.01.

To characterize the proliferation features of our myeloma PDX, we recorded the time it took for the tumor to grow from the inoculation to the diameter of 6-8 mm. The latency period of tumor growth in our myeloma PDX in the generation 0 was significantly longer than that of MM.1S (**Figure 2A**). The growth rate of the MM PDX gradually increased from the generation 0 to 3, which usually reached a steady growth rate after three generations (**Figure 2A**). We also analyzed the cell cycle of myeloma cells. The results showed that there were more PDX tumor cells in the G0/G1 phase while fewer in the G2/M phase than that of MM.1S cells (**Figure 2B**). It is well known that oncogene c-Myc has a critical role in the pathophysiology of MM, which promotes the cell cycle progression and cell growth (27–29). So, we examined the levels of c-Myc protein in MM.1S and PDX tumor cells, and the result showed that PDX tumor cells expressed a lower level of c-Myc protein compared with MM.1S cells (**Figure 2C**). The data suggest that tumor cells from the PDX are more quiescent and less proliferative than MM.1S cells.

### Myeloma PDXs preserve their original drug sensitivities as seen in the clinic

**Figure 3:**
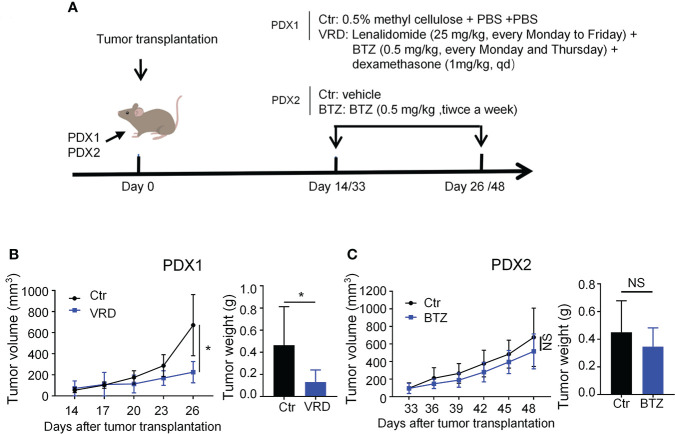
Newly established myeloma PDXs retained their original drug sensitivity and resistance as seen in the clinic. **(A)** Experimental design: *NDG* mice were subcutaneously implanted with tumor pieces (1-2 mm^3^) of PDXs on the flank. When tumors reached 4-6 mm in diameter, mice were randomly divided into two groups. PDX1 models were treated with the VRD regimen or vehicle control. VRD regimen therapy: tumor-bearing mice (n=4-5 every group) received bortezomib (0.5 mg/kg) intraperitoneally every Monday and Thursday, lenalidomide (25 mg/kg) by intragastric administration every Monday to Friday, and dexamethasone (1 mg/kg) intraperitoneally every day for twelve days, and control group was intragastrically administrated with 0.5% methyl cellulose and phosphate-buffered saline (PBS) intraperitoneally. PDX2 models were treated with bortezomib or vehicle control. Bortezomib therapy: tumor-bearing mice (n=3-4 every group) received bortezomib (0.5 mg/kg) intraperitoneally every Monday and Thursday for a total of 5 doses, and the control group was treated with PBS. **(B, C)** Tumor size was measured every 3 days, and tumor weight was measured at the end of the treatment. Ctr, the control group; VRD, VRD treatment group; BTZ, bortezomib treatment group; PDX, patient-derived xenograft; *NS*, no statistically significance. Data were from one experiment representative of two independent experiments with similar results. Data were shown as mean ± SD. Significance was determined by unpaired two-tailed Student’s *t*-test. *NS*, no significance, *P < 0.05.

To test whether the PDXs could recapitulate their drug response features as seen in the clinic, we randomly selected one PDX, named PDX1, to verify its therapeutic sensitivity to VRD (bortezomib + lenalidomide + dexamethasone) regimen, the first-line therapy of MM. PDX1 was derived from a newly diagnosed patient (No. 1) who achieved complete remission after four-cycle treatment of VRD (**Table S1**). We treated PDX1 with the VRD regimen, and the data showed that VRD therapy significantly inhibited the tumor growth of PDX1 (**Figures 3A, B**). Then we chose another myeloma PDX, named PDX2, which was derived from a MM patient resistant to bortezomib therapy in the clinic (**Table S1**). Bortezomib was intraperitoneally administered with a dose of 0.5 mg/kg twice a week in our PDX2 according to previous studies (30, 31). After 5 doses of bortezomib treatments, there was no significant difference of the tumor growth of PDX2 betweent bortezomib and vehicle control group (**Figures 3A, C**). The data suggest that PDX2 retains its original drug resistance. The similar drug sensitivity between the patients and their derived PDXs suggests the feasibility to use these established PDX models to conduct translational myeloma research.

### Anlotinib treatments suppress tumor growth of myeloma PDXs and induce cell apoptosis even in the bortezomib-resistant model

**Figure 4:**
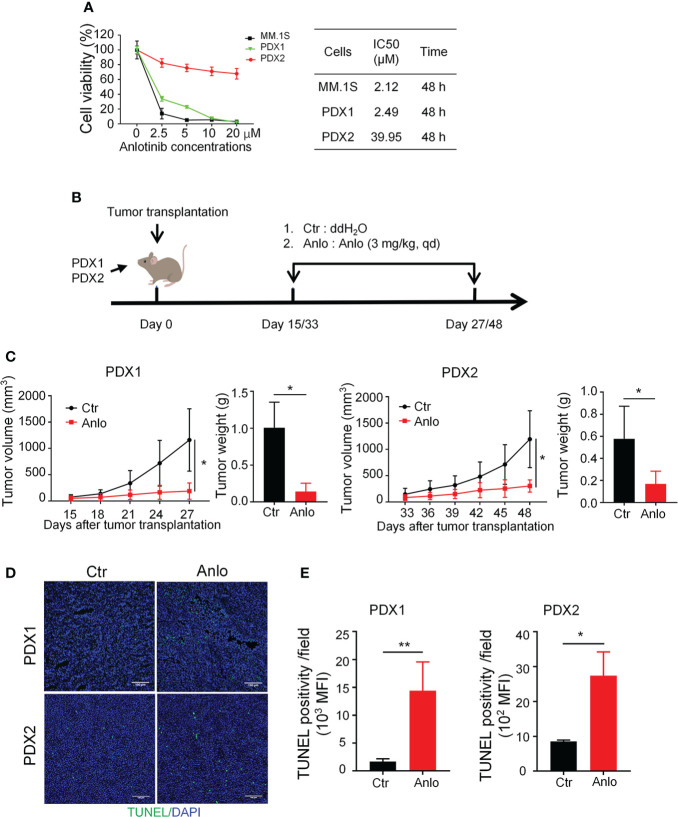
Anlotinib therapy significantly suppressed the growth of myeloma PDXs and induce cell apoptosis even in the bortezomib-resistant model. **(A)** Single-cell suspensions of tumor tissues from PDX1 and PDX2 were prepared. CD138^+^ myeloma cells were purified from the single-cell suspension of PDXs by Human CD138 MicroBeads. MM.1S and CD138^+^ myeloma cells from PDX1 and PDX2 were treated with anlotinib (0-20 μM) for 48 h, and the cell viability was analyzed by CCK8 assays. Each experiment was performed in triplicate. The IC50 was calculated based on the dose-response curve using SPSS version 23 software. **(B)** Experimental design: *NDG* mice were subcutaneously implanted with tumor pieces (1-2 mm^3^) of PDXs on the flank. When tumors reached 4-6 mm in diameter, mice were randomly divided into two groups.The anlotinib group (n=5-6 every group) was administered intragastrically with anlotinib (3 mg/kg) daily for 12 or 15 days, and the control group was treated with double distilled H_2_O (dd H_2_O). **(C)** Tumor size was measured every 3 days, and tumor weight was measured at the end of the treatment. **(D)** Tumors were isolated and stained with TUNEL and DAPI. Representative images showed apoptotic cells. Scale bar: 100 μm. The TUNEL positivity (green) indicated apoptotic cells. **(E)** Quantification of TUNEL-positive cells. PDX, patient-derived xenograft; Ctr, control group; Anlo, anlotinib treatment group; MFI, mean fluorescence intensity. Data were shown as mean ± SD. Significance was determined by unpaired two-tailed Student’s *t*-test. *P < 0.05, **P < 0.01.

Previous studies of our research team have reported the anti-MM activity of anlotinib *in vitro* and *in vivo* (19), thus we would like to assess the efficacy of anlotinib therapy in our newly established MM PDXs. Firstly, we treated PDX1 and PDX2 tumor cells with different concentrations of anlotinib (0-20 μM) *in vitro*. Anlotinib induced dose- and time-dependent cytotoxicity in PDX1 and MM.1S tumor cells with IC50 values of 2.49 and 2.12 μM at 48 h, while in PDX2 tumor cells, anlotinib only exerted slight dose-dependent cytotoxicity at 48 h with a higher IC50 value 39.95 μM (**Figure 4A**). Therefore, PDX1 tumor cells are much more sensitive than bortezomib-resistant PDX2 tumor cells to anlotinib treatments *in vitro*.

Considering the differential cytotoxicities of anlotinib in PDX1 and PDX2 tumor cells *in vitro*, we next evaluated the *in vivo* effects of anlotinib treatments in PDX1 and PDX2. Firstly, we detected the dose effects of anlotinib treatments in MM.1S myeloma. The MM.1S tumor-bearing *NDG* mice were treated with 1.5, 3.0, or 6.0 mg/kg anlotinib for four doses (**Figure S1A**). Anlotinib treatments at 3.0 mg/kg and 6.0 mg/kg showed similar tumor growth inhibition (**Figure S1B**). Thus, we chose 3.0 mg/kg as the treatment dosage of anlotinib. After exposed to either 3.0 mg/kg anlotinib or vehicle control for 12 or15 days in PDX1 or PDX2, respectively, the rates of tumor growth inhibition (TGI) upon anlotinib treatments were 77.78% in PDX1and 55.39% in PDX2 (**Figures 4B, C**). These data show that anlotinib exhibits potent anti-MM activity even in bortezomib-resistant PDX *in vivo*.

Furthermore, we analyzed the influence of anlotinib treatments on cell apoptosis in MM PDXs. The TUNEL stainings showed that the amount of apoptotic cells was significantly increased in anlotinib-treated PDX tumors compared with vehicle control groups (**Figures 4D, E**). Moreover, the anlotinib group had 8.56 times as many apoptotic cells as the control group in PDX1, while it was only 3.20 times in PDX2 (**Figures 4D, E**).

### Anlotinib treatments suppress tumor angiogenesis in myeloma PDXs

**Figure 5:**
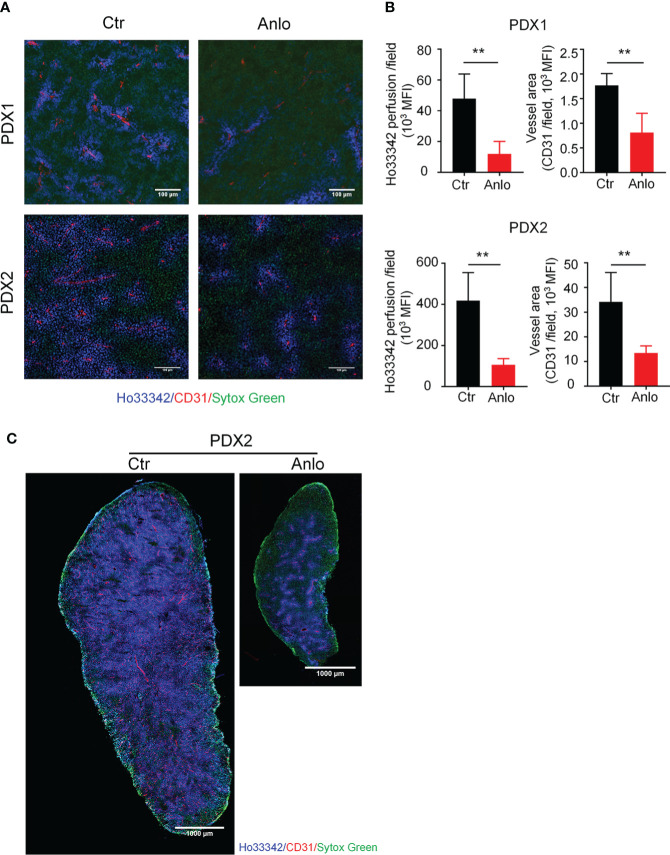
Anlotinib therapy suppressed tumor angiogenesis in MM PDXs. Tumor-bearing *NDG* mice were prepared and treated as described in **Figure 4**. Mice were intravenously injected with 200 μg Ho33342 five minutes before tumor harvest. Tumor tissues were sectioned and stained with anti-CD31 antibody. **(A)** Representative images indicated tumor vessel density (red) and tumor vessel perfusion (blue) in myeloma PDX1 and PDX2. Scale bar: 100 μm. **(B)** Quantification of tumor vessel density and tumor vessel perfusion. **(C)** Representative images of whole tumor tissues showed CD31 staining and Ho33342 perfusion in PDX2. Scale bar: 1000 μm. PDX, patient-derived xenograft; Ctr, control group; Anlo, anlotinib treatment group; MFI, mean fluorescence intensity; CD31, an endothelial cell marker. Sytox green (green): cell nuclei. The intensity of Ho33342 perfusion reflects tumor vascular function. Data were from one experiment representative of two independent experiments with similar results. Data were shown as mean ± SD. Significance was determined by unpaired two-tailed Student’s *t*-test. **P < 0.01.

As a novel multi-targeted receptor tyrosine kinase inhibitor that inhibits pro-angiogenic signaling pathways, the anti-tumor effects of anlotinib in solid tumors are largely attributed to the suppression of tumor angiogenesis (32–35). To determine the effect of anlotinib treatments on myeloma angiogenesis, we evaluated tumor vessel density and tumor vessel perfusion in myeloma PDXs. In the PDX1 and PDX2 models, both tumor blood vessel density and perfusion were decreased in the anlotinib-treated group compared with the vehicle control group, suggesting that anlotinib treatments suppressed tumor vascular function (**Figures 5A, B**). Considering the high heterogeneity of tumor microenvironment (TME) and the uneven distribution of tumor blood vessels (36, 37), we next took the whole images of the cross-sections of tumor tissues in the PDX2 model. Consistent with the previous results, tumor vascular function of myeloma PDX2 was also significantly decreased after anlotinib treatments (**Figure 5C**). To determine whether anlotinib treatment influences the vascular function of normal tissues, we examined blood vessels in colon tissues and found that vascular function in colon tissues was not affected by anlotinib treatments (**Figure S2**). These results demonstrated that anlotinib treatments suppressed tumor angiogenesis without the effect on normal tissue vascular function in myeloma PDXs.

### Anlotinib treatments in myeloma PDXs increase the proportion of tumor-associated macrophages and polarize TAMs from an M2- to an M1-like phenotype

**Figure 6:**
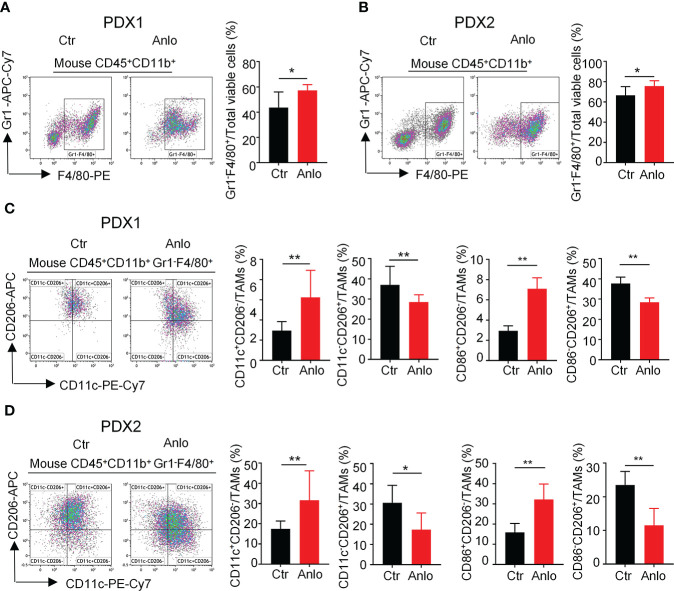
Anlotinib treatments promoted the polarization of tumor associated macrophages (TAMs) from an M2- to an M1-like phenotype in myeloma PDXs. Tumor-bearing *NDG* mice were prepared and treated with anlotinib or vehicle control as described in **Figure 4**. Tumors at the end of treatments were excised and the single cell suspensions were prepared for flow cytometry. The aggregated events and dead cells were gated out. **(A, B)** Representative images and the quantification of tumor-associated macrophages (TAMs) in myeloma PDX1 and PDX2. **(C, D)** Representative images and the quantification of TAM subsets in myeloma PDX1 and PDX2. TAMs: CD45^+^CD11b^+^Gr1^-^F4/80^+^, CD11c^+^CD206^-^: CD45^+^CD11b^+^Gr1^-^F4/80^+^CD11c^+^CD206^-^, CD11c^-^CD206^+^: CD45^+^CD11b^+^Gr1^-^F4/80^+^CD11c^-^CD206^+^, CD86^+^CD206^-^: CD45^+^CD11b^+^Gr1^-^F4/80^+^CD86^+^CD206^-^, CD86^-^CD206^+^: CD45^+^CD11b^+^Gr1^-^F4/80^+^CD86^-^CD206^+^. PDXs, patient-derived xenografts; Ctr, control group; Anlo, anlotinib treatment group. Significance was determined by unpaired two-tailed Student’s *t*-test. Data were from one experiment representative of two independent experiments with similar results. All data were presented as means ± SD. * *P* < 0.05, ** *P* < 0.01.

**Figure 7:**
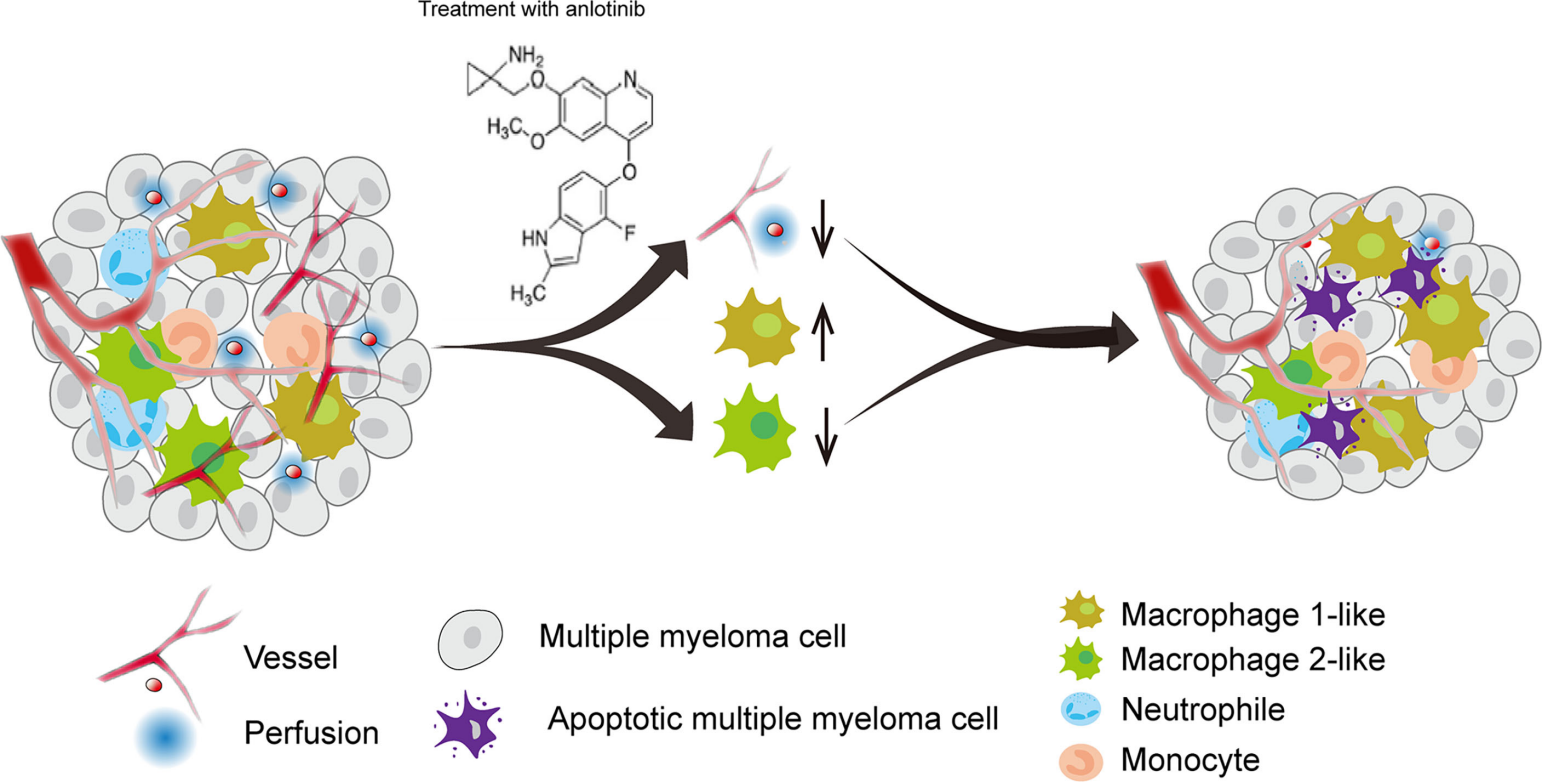
A model diagram of anlotinib therapy in MM. Anlotinib may exert anti-MM effects by inhibiting tumor angiogenesis and polarizing TAMs from an M2-like to an M1-like phenotype to induce cell apoptosis.

Tumor angiogenesis and tumor immunity are two hallmarks of TME. Moreover, the immune-vascular crosstalk in TME plays a critical role for tumor angiogenesis (38). To investigate how anlotinib treatments exert the anti-agiogenesis effect, we analyzed the impacts of anlotinib on the tumor immunity of PDXs. The flow cytometric analysis showed that anlotinib treatments increased the proportions of TAMs (CD45^+^CD11b^+^Gr1^-^ F4/80^+^) compared with that of control groups in both PDX1 and PDX2 models (**Figures 6A, B**). TAMs are very plastic with a continuum of phenotypes, of which M1- and M2-like TAMs represent two extreme phenotypes exhibiting antitumoral or protumoral effects, respectively. CD11c and CD206 are markers commonly used to identify M1- *vs.* M2-like TAMs (39, 40). Further analysis of the subpopulations of TAMs showed that the rates of M1-like (CD45^+^CD11b^+^Gr1^-^F4/80^+^CD11c^+^CD206^-^) TAMs were elevated while the rates of M2-like (CD45^+^CD11b^+^Gr1^-^F4/80^+^CD11c^-^CD206^+^) TAMs were reduced in anlotinib-treated groups, compared with vehicle control groups in both PDX1 and PDX2 models (**Figure 6B**). CD86 is another acknowledged maker of M1-like TAMs (41, 42), we further confirmed the rise of M1-like TAMs and the decline of M2-like TAMs by CD86 after anlotinib treatments in PDX1 and PDX2 (**Figures 6C, D**). Therefore, these data show that anlotinib treatments promote the accumulation of antitumoral M1-like TAMs in MM PDXs. Altogether, our data suggest that anlotinib treatments inhibit tumor angiogenesis, facilitate the polarization of TAMs from an M2- to an M1-like phenotype, and induce cell apoptosis to inhibit the growth of myeloma PDXs (**Figure 7**).

## Discussion

Currently, MM remains an incurable malignant disease with a high risk of relapse and resistance. To explore novel therapeutic strategies to overcome drug resistance and improve the efficacy of myeloma treatments, we established a novel and more practical myeloma PDX, including bortezomib-resistant PDX. Our newly established MM PDXs not only preserved the essential cellular features of MM cells, but also recapitulated their original drug sensitivities as seen in the clinic. By using these MM PDX models, we found that anlotinib treatments significantly suppressed the growth of MM in both newly diagnosed and bortezomib-resistant PDXs. Anlotinib could facilitate the polarization of TAMs from an M2- to an M1-like phenotype, inhibit tumor angiogenesis, and promote cell apoptosis in PDX tumors. These findings suggest that anlotinib is a promising drug to treat MM and may overcome bortezomib resistance. This study also suggests that subcutaneous myeloma cell inoculation following series transplantation in *NDG* mice is a feasible and practical way to develop myeloma PDX for myeloma study.

In this study, we successfully established myeloma PDXs through subcutaneous inoculation of primary mononuclear cells from MM patients in *NDG* mice. These primary mononuclear cells took a couple of months to adjust to the subcutaneous microenvironment and then started to grow stably in *NDG* mice. But not all primary mononuclear cells from MM patients are able to develop tumors by subcutaneous inoculation in *NDG* mice. Three of our PDXs are from BM samples, and another one is from the pleural effusion sample of relapsed and resistant MM patient. Extramedullary plasmacytoma in MM patients is generally developed at the relapse and refractory stage, which become more aggressive and independent of the BM microenvironment, infiltrate other organs or circulate in the peripheral blood (43). Thus, the capability of myeloma cells to grow outside of the BM microenvironment possibly indicates their aggressiveness. Lourdes Farre et al. reported a patient-derived orthotopic xenograft generated from an extramedullary myeloma patient with a cutaneous lesion by subcutaneously implanting tumor tissue (44). From this perspective, subcutaneous myeloma PDX might allow more aggressive myeloma clones to grow up, paving a new avenue to investigate the mechanisms underlying the drug-resistance and explore novel treatment regimens.

Tumor cells from our myeloma PDX expressed CD138 and CD38 with a mature plasma-like morphology and atypic nucleus. Importantly, light chain lambda was restrictively expressed on most tumor cells, which suggested that these tumor cells probably originated from the same clonogenic population. The cell proliferative activity of our myeloma PDX was relatively poorer than that of MM.1S cells, which might because that MM.1S cells have been cultured on plastic over decades and obtained a reproductive advantage. However, these tumor cells from PDXs have distinctive advantages compared to immortalized myeloma cell lines. Owing to that immortalized cell lines are limited in number and diversity, they cannot imitate the complexity of human tumors and only provide explicit insights into human disease (45). Tumor PDXs can more closely reproduce patient tumor behavior and maintain clonal diversity than other models based on the injection of cell lines (46). Recent studies have reported that zebrafish is an available model to establish MM PDX, which permits rapid growth of human MM cells and can be used to investigate the cytotoxicity of compounds (47–49). However, the MM PDX zebrafish model is not suitable for studying tumor blood vessels. Although our myeloma PDX model didn’t completely mimic typical myeloma manifestations, such as osteolysis, hypercalcemia, anemia and renal function damage, it retained primary drug sensitivity as seen in the clinic and is suitable to study tumor blood vessels. What’s more, our PDXs can be passaged and amplified by a series of tumor tissue transplantations. Hence, subcutaneous myeloma PDX can be an important preclinical PDX model to explore new therapeutic strategies.

Our study discovered that PDX1 and bortezomib-resistant PDX2 tumors in different states show distinct sensitivity to anlotinib therapy. *In vitro*, PDX1 tumor cells could be markedly suppressed by anlotinib. The directly cytotoxic mechanism of anlotinib on MM deserves further study. Cao Y et al. found that C-Myc as a direct target contributes to the anti-MM effect of anlotinib (19). Gangyang Wang et al. found that anlotinib suppresses the phosphorylation of MET and the downstream signaling pathway activation in osteosarcoma (34). While bortezomib-resistant PDX2 tumor cells only show mild response to the direct cytotoxicity of anlotinib *in vitro*. The difference in drug sensitivity may be due to the origin of PDX2 tumor cells, which is generated from a patient with relapsed, resistant, and extramedullary MM.

Previous reseaches have found that anlotinib suppress the growth of tumors by reducing tumor angiogenesis *via* targeting VEGFR, PDGFR and FGFR related signaling pathways (32, 33). Interestingly, our study demonstrates that anlotinib treatment increases the percentages of TAMs and polarizes TAMs from an M2- to an M1-like phenotype. M2-like macrophages express more pro-angiogenic factors than the M1-like subset (50). In addition, pro-angiogenic macrophages resemble an M2-like phenotype in the tumor microenvironment (51–53). Hence, anlotinib treatment may reduce tumor angiogenesis by TAM polarization in myeloma, the underlying mechanism needs to be further studied.

Anlotinib therapy may exert anti-MM activities *via* direct cytotoxicity and/or suppressing tumor angiogenesis. *In vitro*, anlotinib exhibits direct cytotoxicity against tumor cells. Whereas *in vivo*, anlotinib could exert anti-MM effect through direct cytotoxicity and anti-angiogenesis. Considering the differential responses of resistant PDX2 tumors *in vitro* and *in vivo*, the growth of PDX2 tumors could be suppressed mainly by the anti-angiogenesis activity of anlotinib. Indeed, the apoptosis rate of anlotinib in PDX1 is higher than that in PDX2. It indicates that in PDX1, the apoptosis might result from the direct cytotoxic and anti-angiogenesis effect of anlotinib, while in resistant PDX2, the apoptosis merely result from anti-angiogenesis effect. Therefore, when myeloma cells are in the BM microenvironment or have infiltrated other organs, we speculate that anlotinib could suppress myeloma through direct cytotoxicity and anti-angiogenesis activity. However, anlotinib could only exert direct cytotoxicity against myeloma cells when they diffuse into the peripheral blood. Therefore, the anti-MM efficacy of anlotinib may be stronger in the BM and other organs than in the peripheral blood.

## Conclusions

In summary, myeloma PDX can be successfully established by subcutaneous inoculation in *NDG* mice following series tumor tissue transplantations. Tumor cells from myeloma PDX retain the essential cellular characteristics of MM and preserve their original therapeutic sensitivities as seen in the clinic. Strikingly, anlotinib therapy suppresses the growth of resistant MM. Furthermore, anlotinib treatments suppress tumor angiogenesis, promote the M1-polarization of TAMs, and induce cell apoptosis in MM PDXs. Taken together, subcutaneous myeloma PDX is a practical preclinical model to explore novel therapies for MM, and anlotinib could be served as a novel promising agent for relapsed and resistant MM.

## Data availability statement

The original contributions presented in the study are included in the article/**Supplementary Material**. Further inquiries can be directed to the corresponding authors.

## Ethics statement

The studies involving human participants were reviewed and approved by The Ethics Committee of The Third Affiliated Hospital of Soochow University, The First People’s Hospital of Changzhou. The patients/participants provided their written informed consent to participate in this study. The animal study was reviewed and approved by The Ethics Committee of The Third Affiliated Hospital of Soochow University, The First People’s Hospital of Changzhou. Written informed consent was obtained from the individual(s) for the publication of any potentially identifiable images or data included in this article.

## Author contributions

YHY, YHH, and WYG designed the experiments. YHY, YC, FW, FL, YTG, YLin, YLiu, and WMD collected clinical samples and analyzed the data. TBC performed HE and IHC staining. YHY, XYM, PF, LQ, and SXG conducted the experiments. YHY, YC, and PF analyzed the data and generated the figures. YHY and YHH wrote the manuscript. YHH and WYG supervised the project. All authors read and approved the final manuscript.

## Funding

This work was supported partly by the Foundations of Changzhou Sci&Tech Program (No. CJ20200118, CJ20210075), the key project of Jiangsu Provincial Health Commission (NO. ZD2021043), the Guiding Project of Changzhou Health Bureau (NO. WZ201708), the Young Talent Development Plan of Changzhou Health Commission (CZQM2020023), and the Collaborative Innovation Center of Hematology, and the Priority Academic Program Development of Jiangsu Higher Education Institutions.

## Acknowledgments

We would like to thank Naidong Zhang and Ziwei Qi (all from Soochow University) for their technical support.

## Conflict of interest

The authors declare that the research was conducted in the absence of any commercial or financial relationships that could be construed as a potential conflict of interest.

## Publisher’s note

All claims expressed in this article are solely those of the authors and do not necessarily represent those of their affiliated organizations, or those of the publisher, the editors and the reviewers. Any product that may be evaluated in this article, or claim that may be made by its manufacturer, is not guaranteed or endorsed by the publisher.
