# Supplementary material for: Novel myeloma patient-derived xenograft models unveil the potency of anlotinib to overcome bortezomib resistance
Source: Front Oncol. 2022 Aug 5;12:894279. doi: 10.3389/fonc.2022.894279 (PMC9389337; doi:10.3389/fonc.2022.894279)
Supplement: Supplementary file 1 [file DataSheet_1.docx]

Supplementary Material


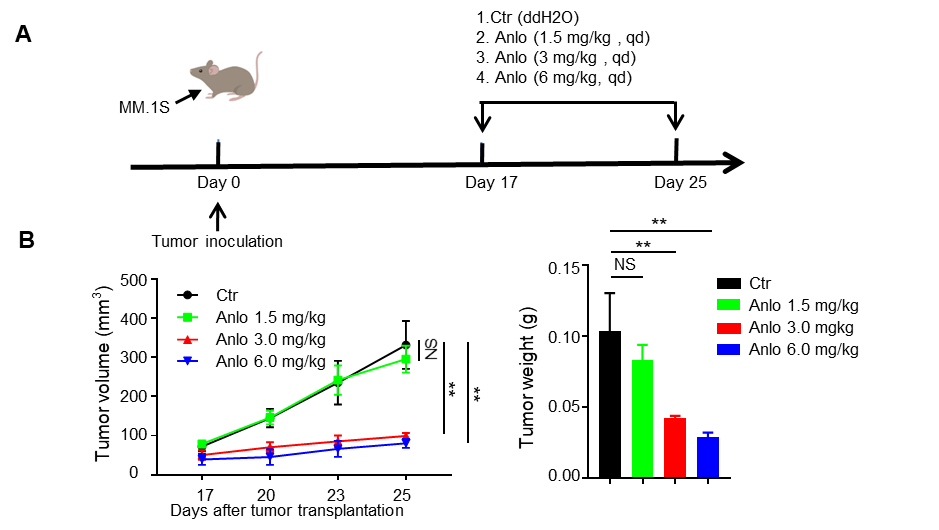


**Supplementary Figure 1.** **Anlotinib therapy inhibited MM.1S tumor growth in a dose-dependent manner.** (A) Experimental design: NDG mice were subcutaneously inoculated with MM.1S cells on the flank. When tumors reached 4-6 mm in diameter, mice were randomly divided into 4 groups and received intragastric administration of anlotinib (1.5 mg/kg, 3 mg/kg, and 6 mg/kg) or vehicle control daily for 9 days. (B) The tumor growth curves and tumor weight of the control group and different doses of anlotinib groups. Ctr: control group treated with ddH_2_O. Anlo: anlotinib treatment groups. *NS*: no statistically significance. Data were shown as mean ± SD. Significance was determined by unpaired two-tailed Student's *t*-test. ***P* < 0.01


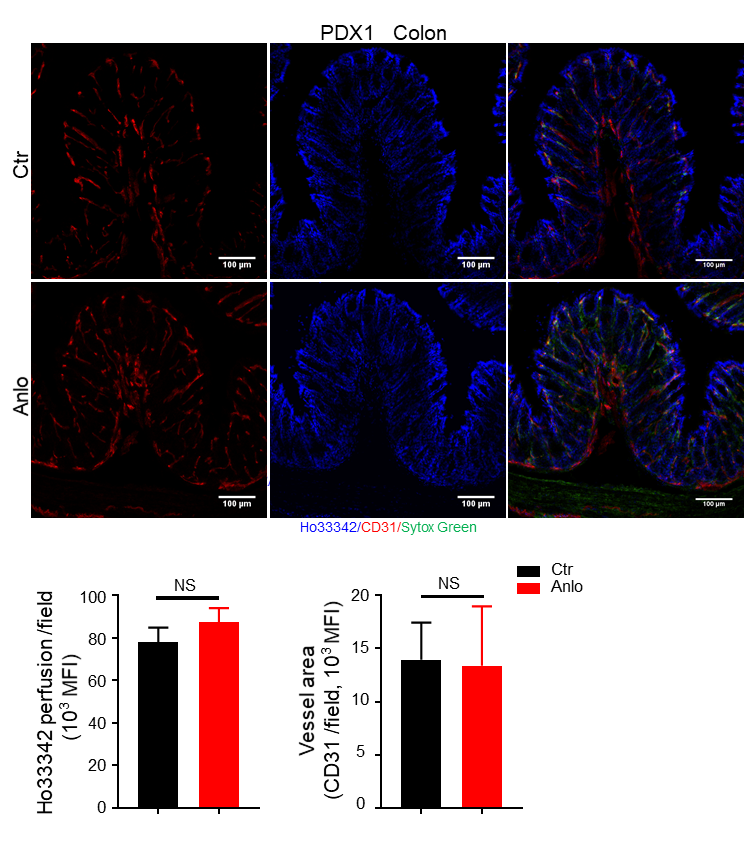


**Supplementary Figure 2.** **Anlotinib therapy did not affect vascular function of murine colon tissues in myeloma PDX.** Tumor-bearing NDG mice were treated as described in Fig. 4. The cross-sections of colon tissues were captured by a confocal microscopy. The mean fluorescence intensity (MFI) of vessel density and Ho33342 perfused area over the colon tissues were quantified. Scale bar: 100 μm. Sytox green (green): cell nuclei, Ho33342 (blue): blood vessel perfusion, and CD31 (red): endothelial cells. PDX: patient-derived xenograft. Ctr: control group. Anlo: anlotinib treatment group. *NS*: no statistically significance. Data were shown as mean ± SD. Significance was determined by unpaired two-tailed Student's *t*-test.
