# Supplementary material for: Novel myeloma patient-derived xenograft models unveil the potency of anlotinib to overcome bortezomib resistance
Source: Front Oncol. 2022 Aug 5;12:894279. doi: 10.3389/fonc.2022.894279 (PMC9389337; doi:10.3389/fonc.2022.894279)
Supplement: Supplementary file 2 [file Table_1.docx]

| No. | Age (years) | sex | Disease stage | DS | R-ISS | Type | Treatments | Cytogenetics/FISH | Bone marrow biopsy and immunohistochemical staining | Bone marrow cell morphology | Flow cytometry of bone marrow cells |
| --- | --- | --- | --- | --- | --- | --- | --- | --- | --- | --- | --- |
| P1 | 48 | M | Diagnosis | IIA (without bone destruction) | I | IgG-kappa | VRDx4, AutoSCT,  Bortezomib/ Lenalidomide /Dexamethasone | 57，XY, +1, +2, +3, +5, dup(6)(p25p12), dup(6)(p12p27), del(8) (p12p23), dup(8)(q11q24), +9, dup(10)(p15p24), dup(10) (q25q26), +11, +12, +15, +18, +19, dup(21)(q11q21), +22 | Abnormal plasma cell rate 30%, dominated by immature plasma cells  Immunohistochemical staining of abnormal plasma cells: CD38^+^, CD138^+^, cKappa^+^, CD56^+^, Bcl-2^+^, MUM1^+^ (part), cLambda^-^, CD20^-^, PAX5^-^, CD3^-^, CD5^-^, CyclinD1^-^, CD117^-^, C-MYC^+^ (5%-10%), Ki-67^+^ (10%-20%) | Plasma cell rate 12.5%，occasionally appearing immature plasma cells and binuclear plasma cells. | 8.33% of bone marrow nucleated cells were a clonal plasma cell population, which positively expressed cKappa, CD56, CD138, CD38, CD81 and CD45 (+/-), and negatively expressed cLambda, CD19, CD117, and CD27. |
| P2 | 71 | M | Relapse | IIIB (without bone destruction) | III | IgD-lambda | VDx4, VTDx4, VCDx2, IADx2, | 1q21 amplification (40%); the deletion of Rb1 (40%), the deletion of 13q14 (44%), IgH rearrangement (30%) | Pleural effusion pathology: small heterotypic cells. Immunohistochemical staining of abnormal plasma cells：AE1/AE3^-^, CD20^-^, CD79a^-^, CD38^+^, CR^-^. | Plasma cell rate 19% (diagnosis) and 35% (relapse). | 16.5% of bone marrow nucleated cells were a clonal plasma cell population and positively expressed CD138, CD38 and cLambda. |
| P3 | 59 | M | Diagnosis | IA (with bone destruction) | I | IgG-lambda | VRDx4, AutoSCT | 1q21 amplification (80%), the deletion of 13q14 (-), Rb1 rearrangement (-), P53 rearrangement (-), IgH rearrangement (-), and t（4；14）/ t（14；16）(-) | Abnormal plasma cell rate 5%-10%, dominated by mature plasma cells. Immunohistochemical staining of abnormal plasma cells: CD38^+^, CD138^+^, cLambda^+^, MUM1^+^ (part), Bcl-2^+^ (part), CD56^+^ (part), cKappa^-^, CD20^-^, PAX5^-^, CD3^-^, CD5^-^, CyclinD1^-^, CD117^-^, Bcl-6^-^, C-MYC^+^ <5% | Plasma cell rate 16.5%，occasionally appearing binuclear plasma cells. | 15.5% of bone marrow nucleated cells were a clonal plasma cell population, which positively expressed cLambda, CD19, CD56, CD27, CD81, CD138, CD38, and CD45(+/-), and negatively expressed cKappa and CD117. |
| P4 | 44 | F | Diagnosis | IIB (with bone destruction) | II | IgG-kappa | VAD, ICD, AutoSCT | 1q21 amplification (75%); the deletion of 13q14 (79%), the deletion of Rb1 (80%), the deletion of P53 (-), IgH rearrangement (85%) | Abnormal plasma cell rate >80%，dominated by immature plasma cells. Immunohistochemical staining of abnormal plasma cells: CD38^+^, CD138^+^, cKappa^+^，MUM1^+^，Bcl-2^+^，CD56^+^ (part)，cLamdda^-^, CD20^-^, PAX-5^-^, CD3^-^, CD5^-^，CyclinD1^-^, CD119^-^, C-MYC^+^ <5%，Ki-67^+^ < 5%. | Plasma cell rate 45.5%，immature plasma cell rate 18%. | 11.2% of bone marrow nucleated cells were a clonal plasma cell population, which positively expressed cKappa, CD56, CD138, CD38(++), CD81, CD27(+/-) and CD45(+/-), and negatively expressed cLambda, CD19, and CD117. |

Table S1 The characteristics of four patients.

AutoSCT: Autologous hematopoietic stem cell transplantation; F: female; IAD: ixazomib + liposomal doxorubicin + dexamethasone; ICD: ixazomib + cyclophosphamide + dexamethasone; M: male; P1-4: patient1-4 correspond to PDX1-4; VAD: bortezomib + doxorubicin + dexamethasone; VCD: bortezomib + cyclophosphamide + dexamethasone; VD: bortezomib + dexamethasone; VTD: bortezomib + thalidomide + dexamethasone; VRD: bortezomib + lenalidomide + dexamethasone.
